# Rigorous evaluation of a substance use and teen pregnancy prevention program for American Indian girls and their female caregivers: a study protocol for a randomized controlled trial

**DOI:** 10.1186/s12889-021-11131-x

**Published:** 2021-06-21

**Authors:** Rachel A. Chambers, Jaime Begay, Hima Patel, Jennifer Richards, Danielle Nelson, Summer Rosenstock, Ronni Huskon, Kristin Mitchell, Tiffani Begay, Lauren Tingey

**Affiliations:** Johns Hopkins Center for American Indian Health, Johns Hopkins Bloomberg School of Public Health, 415 North Washington Street, Baltimore, MD 21231 USA

**Keywords:** Primary prevention, Intergenerational, Teen pregnancy prevention, Substance use prevention, Female, Native American, Culture

## Abstract

**Background:**

Early sexual initiation is associated with higher risk for sexually transmitted infection, teen pregnancy, domestic violence and substance use in later adolescence and early adulthood. Native American adolescents are more likely to have early sexual initiation compared to other racial/ethnic groups. Few programs designed with and for Native adolescents to delay sexual initiation and substance use have been tested through rigorous evaluations. This is the protocol for the randomized controlled trial of the Asdzáán Be’eena’ program, a teen pregnancy and substance use prevention program for young Native girls and their female caregivers.

**Methods:**

*N* = 410 female adolescents ages 10–14 and their female caregivers will be enrolled in the study and randomized to the intervention or control arm. The intervention consists of the 11-session Asdzáán Be’eena’ program. The control arm consists of mailed non-monetary incentives. All participants will complete evaluations at baseline and 3 follow-up timepoints (immediate, 6 and 12 months post intervention). Evaluations include measures to assess protective factors associated with delayed sexual initiation and substance use.

**Discussion:**

This is one of the first rigorous evaluations of a gender-specific, culturally tailored teen pregnancy and substance use primary prevention program for Native girls and their female caregivers. If proven efficacious, Native communities will have a culturally appropriate program for promoting protective factors associated with delayed substance use and sexual risk taking.

**Trial registration:**

NCT04863729; April 27, 2021.

## Introduction

### Background and rationale

Extensive research documents how both early sexual debut and substance use intiation adversely impact the health of an individual. Specifically, early sexual debut increases the likelihood of poor sexual and reproductive health outcomes, such as sexually transmitted infections (STI) and unintended teen pregnancy [1–4]. It is also associated with a greater number of sexual partners and increased risk of being a victim of intimate partner violence [4–6]. In terms of substance use, early initiation of substance use adversely impacts the cognitive and emotional growth and development of adolecents [7–9].

Early sexual debut and substance use are also costly for the individual, society and tax payer. A recent analysis conducted by Rotz et al. found large economic savings of preventing teen sexual activity and substance use. The analysis estimates a net benefit of up to $52,109 for females and $27,861 for males from delaying voluntary sexual activity to age 15 or older [10]. For substance use, the estimated cost savings for preventing underage drinking is estimated to be up to $12,313 for individuals [10].

Not surprisingly, substance use initiation is one of the most significant risk factors for sexual initiation and unprotected sex among adolescents. In fact, these behaviors tend to co-occur, particularly among more vulnerable subgroups of adolescents [11, 12]. These behaviors also share similar risk factors including lack of family cohesion [13–17] and poor adolescent functioning (having high levels of internalizing and externalizing behaviors). They also share many protective factors including connection to culture [18–21], parent-child relationship, self-efficacy and future aspirations [21–26]. Programs targeting these shared risk and protective factors may have a dual impact: reducing both sexual risk taking and substance use among adolescents.

National data show Native American (Native) adolescents are more likely to initiate sex before age 13 than all other U.S. racial/ethnic groups, except African American adolescents [27]. Native high school youth are also more likely to have multiple sex partners and to ever have had sex compared to youth of all races [27, 28]. Native youth also experience higher rates of STIs and teen pregnancy than their non-Native counterparts; in 2018, U.S. national Chlamydia and Gonohrrea rates among Native Americans were 3.7 and 4.6 times higher than that among Whites [29]. Further, in 2017, the national teen birth rate among 15–19 year old Native Americans was the highest of all races and ethnicities [30, 31]. It follows that Native adolescents have higher rate of substance use and abuse. Compared to all U.S. adolescents, in 2018, Native adolescents ages 12–17 are more likely to engage in past-month binge drinking (5.7% vs. 4.9%), marijuana use (11.1% vs. 7.4%), and other illicit drug use (12.8% vs. 8.7%) [32].

Given the adverse outcomes of early sexual activity and substance use and the high rates of sexual risk taking and substance use among Native adolescents, prevention efforts to delay these behaviors in Native communities are warranted [33]. These efforts may be more advantageous if delivered during early adolescence (ages 10–14), before a child initiates sex or substance use. Early adolescence is a key developmental period in which children continue to solidify their values and relationships, and learn foundational life skills [34–37]. Hindered growth in these areas during this time period has been linked to poorer health outcomes including early sexual initiation, substance use and abuse in later adolescence and adulthood [33]. Thus, programs that promote values, focus on building healthy relationships and teach life skills such as problem-solving and goal-setting in early adolescence are needed.

As opposed to many Western societies, most Native cultures do not have a self-centered orientation to health, but instead have family- and community-oriented health frameworks [38]. Thus, intergenerational programming may be more culturally appropriate [39] than individual-level programs. In addition to cultural congruency, engaging parents alongside adolescents in programming may be advantageous as they can support and reinforce behaviors [40, 41]. While robust efforts have begun to address behavioral health disparities among Native adolescents, currently there are no intergenerational, efficacious primary prevention programs targeting the dual threat of sexual risk taking and substance use for young Native adolesents [42–44]. This study seeks to fill this gap.

### The current study

The objective of the is to assess the efficacy of a program called Asdzáán Be’eena’ (Female Pathways, AB) for increasing protective factors associated with delayed substance use and sexual risk taking among Native girls in early adolescence (ages 10–14) through a randomized controlled trial (RCT). This paper describes the study protocol.

### Objectives

The study will test the efficacy of the AB program on risk and protective factors associated with delaying adolescent substance use and sexual initiation, as well as actual sexual initiation and substance use. By assessing protective and risk factors as well as behaviors among the adolescent and the caregiver, the study team will be able to assess how each of these are impacted by the intervention, and how these interact with one another to moderate program impact.

Primary research questions include: 1) Is the intervention effective in increasing adolescents’ intention to abstain from sex (intention to abstain was chosen as opposed to sexual initiation as we do not anticipate high rates of sexual initiation for the duration of the follow-up time period); 2) Is the intervention effective in improving caregiver-adolescent relationships. Secondary research questions include: 1) Is the intervention effective in improving adolescent risk and protective factors associated with delayed sexual initiation and/or substance use initiation; 2) Is the intervention effective in improving caregiver risk and protective factors associated with delayed adolescent sexual initiation and/or adolescent substance use initiation; 3) Is the intervention effective in delaying sexual initiation and substance use initiation among adolescents; 4) Is the intervention effective in reducing substance use among enrolled caregivers.

The study team hypothesizes a higher proportion of adolescents in the intervention group will state they intend to abstain from sex in the next year and until they graduate high school compared to the control group at 6- and 12-months post intervention. Additionally, we hypothesize that there will be 1) an increase in caregiver-adolescent communication at 6- and 12-months post intervention as measured by a communication scale adapted from a trial with Ojibwe adolescents, 2) an increase in caregiver-adolescent quality time together at 6- and 12-months post intervention as measured by a quality time with parent scale adapted from a trial with Ojibwe adolescents, 3) an increase in maternal warmth at 6- and 12-months post intervention as measured by the Authoritative Parenting Index, and 4) an increase in caregiver monitoring at 6- and 12-months post intervention as measured by the parental monitoring scale.

### Trial design

**Fig. 1:**
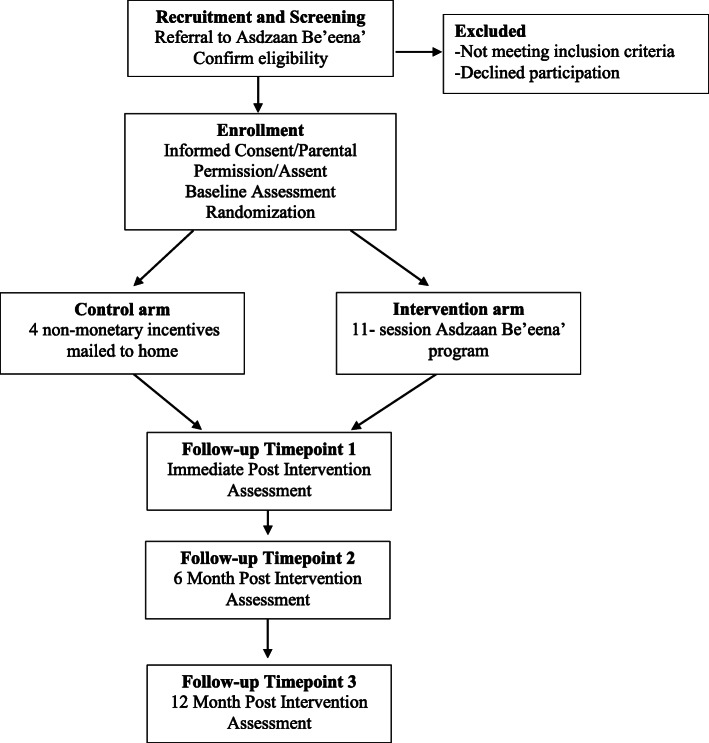
Trial Design

The study is a two-armed randomized controlled trial examining the primary outcomes of adolescent-caregiver relationship, adolescent intention to have sex and adolescent adaptive/maladaptive functioning at 12 months post intervention. The trial is being conducted at two study sites on the Navajo Nation with Navajo girls ages 10–14 and their female caregiver. Randomization will be performed with a stratified block randomization sequence to ensure a 1:1 allocation of study conditions within each of the two study sites Fig. 1.

## Methods/design

### Study setting

The study will be conducted by the Navajo Nation in partnership with Johns Hopkins Center for American Indian Health (Center). The study design was approved by the appropriate Navajo governing bodies including Chapters, Agency Councils and Health Boards as well as the JHU research review board and the Navajo Nation Human Subjects Research Review Board (NNHRRB). This manuscript was approved by the NNHRRB. All deviations from the protocol will be approved by IRBs before being implemented. Once approved, they will be updated in clinicaltrials.gov by the PI. As needed, participants will be reconsenting utilizing consenting documents that include changes to the protocol and have been approved by the appropriate IRBs.

The study is community based intervention and conducted on the Navajo Nation in the United States of America.

#### Study staffing

This study is being conducted at two study sites on the Navajo Nation. The Center has had a physical office and has conducted projects in partnership with the Navajo Nation for over 30 years. Each site has two family health coaches (FHCs), one independent evaluator (IE) and one site coordinator. Other members of the study team, including the principal investigator, program manager, program coordinator and evaluator, are located at the Center’s administrative office in Baltimore, Maryland. The field manager is located in Arizona near the Navajo Nation. All members of the study team are full-time Johns Hopkins employees and are thoroughly trained in human subjects research prior to working with participants. Members of the Baltimore team and the field manager make frequent trips (at least bi-annual) trips to the study sites to conduct training and quality assurance.

#### Family health coaches

FHCs are Navajo women living in one of the study communities. All speak some Navajo. All FHCs will complete extensive (> 60 h) of training in the program curriculum as well as skills necessary to deliver the program including home-visiting, group facilitation, and boundary setting. They will be responsible for delivering the AB program to dyads. They will also be responsible for delivering the non-monetary retention gifts to dyads in the control group.

#### Independent evaluators

Independent Evaluators are Navajo women living in one of the two study communities. All speak some Navajo. All IEs will complete extensive training (> 40 h) in the study protocol including recruitment, consenting and data collection. IEs will recruit participants, administer informed consent, receive parental permission and adolescent assent. IEs will also administer all assessments and provide gift cards for assessment completion.

#### Site coordinators

Site Coordinators are Navajo women living in one of the two study communities. They will provide on-site support to all local study staff.

### Participants and eligibility criteria

Participants are Native female adolescents and their female caregiver (referred to as dyads). Inclusion criteria for the adolescent includes: Native American ethnicity, 10–14 years of age, living within 50 miles of one of the two study sites, and written parental permission and adolescent assent. Participant inclusion criteria for the caregiver includes: Native American ethnicity, ≥ 18 years of age, caregiver to a female child age 10–14 years who will enroll in the study with them, living within 50 miles of a study site, and written informed consent. If the caregiver is not the legal guardian of the adolescent, the adolescent’s legal guardian must agree for the caregiver to participate. To honor cultural views around caregiving, female caregivers who live outside of the home but play a pivotal role in child rearing are welcome to participate. To enroll, both members of the dyad (adolescent and caregiver) must be eligible and enroll. Adolescents in foster care are ineligible to participate.

### Interventions

#### The Asdzaan Be’eena’ program

The AB program is described in detail elsewhere (Chambers 2021). A short description is included here.

#### Program development

Program development occurred from 2016 to 2018 and included the following: 1) 12 focus groups and 15 interviews with Navajo girls ages 8–25, mothers and grandmothers, fathers and grandfathers, as well as traditional practitioners, 2) 10 Community Advisory Board (CAB) meetings each consisting of 8–12 community leaders, 3) administration of a survey to 400 Navajo adult women to assess preferences and experiences with puberty, 4) a three-day intensive workshop with cultural specialists and 5) an extensive review of the literature to identify other programs and requisite risk and protective factors to delay sexual initiation and substance use among Native girls in pre- and early adolescence. Lessons were drafted by the Johns Hopkins curriculum team in consultation with cultural experts, reviewed by the CAB and piloted with *N* = 47 dyads. Evaluation assessments were piloted with participating mothers and daughters at baseline and 3 month time points. Pilot findings indicate the program positively impacted communication between girls and their female caregivers (*p* = 0.2; *p* < .001), improved cultural (*p* = .02) and reproductive health knowledge (*p* = .001), while simultaneously reducing internalizing behaviors (*p =* .001), deviant disorder symptoms (*p* = .04), and attention deficit disorder (*p* = .03) at 3 months post program completion. Female adolescents who completed the program were also more likely to state that they planned to wait to have sex until married (25% vs. 70%, *p* = .218). This preliminary data justified rigorous evaluation of the AB program for impacts on delayed sexual and substance use initiation.

#### Program curriculum

The AB program is a culturally grounded curriculum consisting of eleven lessons organized according to the teachings of the Navajo creation story, designed specifically for Navajo females. Lessons also provide knowledge and skills necessary for delaying substance use and sexual initiation as identified through the focus groups, in-depth interviews and literature review. All lessons are taught by a FHC, and consist of three to five activities designed to teach a skill or provide knowledge about a topic (see table below). At the end of each lesson, 1) the FHC reviews key teachings from the lesson, 2) dyads practice three to five Navajo vocabulary words, 3) the FHC provides the dyad with a positive affirmation, 4) the dyads are given worksheets with information and activities completed during the session, and 5) the FHC gives a scheduling reminder for the next lesson.

#### Program implementation

The AB curriculum is implemented through a mix of individual dyad and group formats (see Table 1). Individual dyad lessons are taught by one FHC to a dyad in their home or another place of their choosing (e.g. the local program office, a local school or clinic). Snacks are provided to dyads during these sessions. Group lessons are taught by 2 FHCs (facilitator and co-facilitator) to groups of 7–12 dyads in a central location in the community (e.g. chapter house, local clinic or school). Meals are provided to dyads during group sessions and transportation to group sessions is provided as needed. Participants complete the program in cohorts of 7–12 dyads. All members of a cohort participate in group lessons together.

**Table 1:** AB Lesson Delivery Format and Content

Lesson number	Format	Topic
1	Individual dyad	Program introduction; discussion of the meaning of the clan system; importance of self-reflection in Navajo culture
2	Individual dyad	Identifying role models; respect for self, others and mother earth
3	Group	Building the caregiver adolescent relationship; strategies for improved communication
4	Individual dyad	Developing support networks; Navajo introductions; family roles and values
5	Group	Navajo history, Navajo teachings related to puberty; Introduction to the female reproductive system
6	Individual dyad	Thinking positively; problem solving skills development
7	Group	Communication styles; dealing with peer pressure; refusal skills
8	Individual dyad	Community and family values; support networks; Kinaaldá (Navajo coming of age ceremony) teachings
9	Group	Reproductive Health 101: puberty and pregnancy; healthy hygiene during puberty; skills to improve caregiver-adolescent communication about sensitive topics Caregiver only: safe sex practices
10	Individual dyad	Self-esteem; identifying personal strengths; goal setting skills
11	Group	Holistic health; promoting others; taking lessons learned forward

#### Control condition

The control condition consists of the delivery of four non-monetary retention items totaling <$20/dyad to the participant’s home. Retention items were selected by the community and provided as a way to continue to stay in contact with families for follow-up data collection.

#### Discontinuing or modifying intervention

Participants will not be allowed to change groups. They will be able to stop participating at the study at any point. Study staff are trained to identify and report adverse events. In the event of an adverse event, the study staff member will call the PI and follow-up with an email detailing the event. All adverse events will be sent to the IRB according to their required schedule. The study will be reviewed by approving IRBs annually. There is no post-trial care or compensation for those who suffer harm from trial participation.

### Recruitment and consent

Participants will be recruited through a combination of non-probability and snowball sampling. First, recruitment fliers will be posted in community gathering spots (i.e., supermarket, community centers, health and human services offices, fitness centers, etc.), on social media (e.g. Facebook) and disseminated during public gatherings through informational booths. Second, caregivers enrolled in the project will be asked to refer other caregivers who may be eligible and interested in the program. Third, IEs will provide information about the study to local agencies that provide services to eligible participants (e.g. Office of Self Reliance, local schools, counseling services etc.) and provide them with referral forms. Independent Evaluators will collect referral forms from these agencies on a regular basis (e.g. weekly). All recruitment materials have contact information for the local project coordinator. To learn about the project and enroll, interested individuals can call the local project coordinator. A study staff member will also reach out to referred caregivers or parents of a referred child. When contact is made with a potential participant, the IE will review a brief script describing the study and assess for eligibility. For those individuals meeting eligibility criteria who express interest in study participation, the study staff member will work with them to set up a time to complete informed consent/parental permission/assent and work with the participant to contact the caregiver who will enroll with them (adolescent and adolescent’s female caregiver). The informed consent process will include a review of how personal information will be collected, shared and maintained to protect confidentiality of the participant’s study data. After both the caregiver and adolescent participants have completed informed consent and adolescent assent respectively, they will complete the baseline assessment and schedule their first lesson.

### Retention

Participants will receive gift cards for participation in assessments ($20 per assessment time point per participant). Additionally, FHCs and IEs will do all they can to keep participants enrolled and engaged including providing transportation to study visits and snacks or meals at these visits. Participants will only be dropped from the program if they request. Outcome data will be collected from all participants regardless of their intervention completion status.

### Outcomes

Assessments will be administered at baseline, immediately following program completion and 6- and 12-months following program completion (see Table 2). Data will be collected in participants’ homes or another private location via a tablet using Research Electronic Data Capture (REDCap). Caregivers will complete all assessments via self-report. Adolescents will complete portions of the outcome assessment asking about sexual and substance use behaviors via self-report. All other portions will be be completed via interview. Participants will be given $20 after completion of the baseline, immediate, six and twelve month post intervention assessments. All measure items were previously piloted with adolescent and caregivers from the community to assess comprehension, language, and cultural relevance. Edits were made accordingly and pilot participants were not eligible for the RCT.

**Table 2:** Study Evaluation: constructs measured, method of administration and timepoint administered

Construct Measured	Completed by	Method ofadministration	Timepoint Administered			
			Baseline	Immediate Post	6 month post	12 month post
**DEMOGRAPHICS**						
Age, gender, language spoken in home, housingsituation and education	Caregiver	Self-Report	X	X	X	X
Age, gender, language spoken in home, housingsituation and education	Adolescent	Interview	X	X	X	X
**PRIMARY AIMS**						
**Caregiver-Adolescent**	Adolescent	Interview	X	X	X	X
**Relationship** [45, 46]						
Quality time together				X		
Communication^a^				X		
Warmth				X		
Monitoring				X		
**Reproductive Health and intentions aboutsexual activity**	Adolescent	Self-Report	X	X	X	X
**SECONDARY AIMS**						
***Adolescent Measures***						
**Individual Level Protective and Risk Factors**						
Self-Regulation [47]	Adolescent	Interview	X	X	X	X
Self-Esteem [47]	Adolescent	Interview	X	X	X	X
Future Aspirations [48]	Adolescent	Interview	X	X	X	X
Perseverance/Resilience [47]	Adolescent	Interview	X	X	X	X
Optimism [47]	Adolescent	Interview	X	X	X	X
Self-Efficacy [47]	Adolescent	Interview	X	X	X	X
Interpersonal Competence [47]	Adolescent	Interview	X	X	X	X
Internalizing and Externalizing behaviors	Adolescent	Interview	X	X	X	X
Reproductive Health Knowledge	Adolescent	Self-Report	X	X	X	X
Self-Regulation [49]	Adolescent	Self-Report	X	X	X	X
Healthy Relationship Knowledge [49]	Adolescent	Self-Report	X	X	X	X
**Cultural Knowledge and Connection**						
Navajo Cultural Knowledge	Adolescent	Interview	X	X	X	X
Participation in Traditional and Religious Activities [50]	Adolescent	Interview	X	X	X	X
Family Engagement in Navajo Culture	Adolescent	Interview	X	X	X	X
**Family**						
Family Conflict, Expressiveness and Cohesion [51]	Adolescent	Interview	X	X	X	X
Parent Expectations and Attitudes [52, 53]	Adolescent	Self-Report	X	X	X	X
**Peer and Trusted Adults**						
Peer Role Models: Youth Asset Survey [48]	Adolescent	Self-Report	X	X	X	X
Peer and Partner Negotiation Skills [49]	Adolescent	Self-Report	X	X	X	X
Communication with Peers [49]	Adolescent	Self-Report	X	X	X	X
Communication with Trusted Adults [49]	Adolescent	Self-Report	X	X	X	X
**Behavioral Intention and Outcomes**						
Substance Use [54]	Adolescent	Self-Report	X	X	X	X
***Caregiver Measures***						
**Mother Daughter Relationship** [45, 46]						
Communication	Caregiver	Self-Report	X	X	X	X
Warmth	Caregiver	Self-Report	X	X	X	X
Monitoring	Caregiver	Self-Report	X	X	X	X
**Individual Level Protective and Risk Factors**						
Parenting Self- Efficacy [55]	Caregiver	Self-Report	X	X	X	X
Parenting Self-Agency [56]	Caregiver	Self-Report	X	X	X	X
Reproductive Health Knowledge	Caregiver	Self-Report	X	X	X	X
Communal Mastery [57]	Caregiver	Self-Report	X	X	X	X
Parent Expectations and Attitudes [52, 53]	Caregiver	Self-Report	X	X	X	X
**Cultural Knowledge and Connection**						
Cultural Connectedness [50]	Caregiver	Self-Report	X	X	X	X
Participation in Navajo practices	Caregiver	Self-Report	X	X	X	X
Experience with Native Language	Caregiver	Self-Report	X	X	X	X
Cultural Knowledge	Caregiver	Self-Report	X	X	X	X
Family engagement with culture	Caregiver	Self-Report	X	X	X	X
**Family**						
Family Conflict, Expressiveness and Cohesion [51]	Caregiver	Self-Report	X	X	X	X
**Behavioral Intention and Outcomes**						
Substance Use [54]	Caregiver	Self-Report	X	X	X	X
**Parent Report on Youth**						
Internalizing and Externalizing behaviors	Caregiver	Self-Report	X	X	X	X
Youth Experience with Substances	Caregiver	Self-Report	X	X	X	X
**OTHER MEASURES REQUIRED BY FUNDER**						
Exit Survey: includes questions about program satisfactionand sexual health behaviors/attitudes [58]	Adolescent	Self-Report		X		

^a^Primary Aim in which sample size was determined

Please see Table 2 for a description of the outcome measures, including method and frequency of administration.

### Data management

All data will be directly entered into REDCap by an intervieweer (IE) or the participant. If assessments are completed via paper, they will be entered by the IE. A data manager will review all data entered into REDCap to ensure completeness and conduct data quality assurance (including checking ranges for variables as applicable). All data will be stored via REDCap, a secure, HIPPA compliant, data collection and storage database. Data will be analyzed part way through the trial by the evaluator. The study management team (including the evaluator, the PI and the program manager) will review data and assess the need to continue the trial.

### Randomization and sample size

This study will use a 1:1 randomized controlled trial design; the unit of randomization will be the individual dyad. Unique participant identification numbers will be used to randomize individual dyads to one of two study groups: intervention or control. Stratified block randomization, using random block size will be used to ensure a 1:1 allocation of study conditions across the two study sites. The sequence will be generated by the program evaluation via STATA and will not be shared with study staff who enroll participants.. Independent Evaluators will enroll participants and reveal study group after the participants complete the baseline assessment. The study assignments are sequentially numbers in sealed envelopes that are only opened at the time of randomization. No blinding will occur.

Sample size and statistical power estimates are predicated on 2 primary hypotheses: 1) that those randomized to the AB intervention will have improved parent-adolescent communication compared to the control group, and 2), that those randomized to the AB intervention group will have a higher proportion of participants who report they intend to abstain from sex.

The sample size of 410 dyads (*n* = 205 per study arm) will be sufficient to detect a 0.3 difference between study groups, with 90% power and 0.05 significance level, on the communication scale at 12 months post-intervention (estimating a mean score (SD) of 2.81(0.85) in the control group). This same sample size will be sufficient to detect an 18% between-group difference, with 80% power and 0.05 significance level, in the proportion of participants who report intention to abstain from sex at 12 months post-intervention (estimating 50% of the control group to report intention to abstain from sex at 12 months post-intervention). This sample size will also be able to detect meaningful differences in other key confirmatory research question outcomes at 12 months post-intervention. The following presents minimum detectable differences between the intervention and control groups with at least 80% power, given the assumptions listed above: 0.39 between-group difference in internalizing behaviors (estimating a mean score (SD) of 5.23 (0.99) in the control group), and 0.25 between-group difference in externalizing behaviors (estimating a mean score (SD) of 2.04 (0.63) in the control group). All estimates assume 10% attrition between enrollment and program completion and a 25% attrition rate between program completion and the 12 month follow-up time point.

### Statistical analysis

The between study group equivalence of participant characteristics and outcome measures at baseline will be compared using Chi-squared tests (dichotomous/categorical data) and t-tests (continuous data). Study hypotheses related to the confirmatory questions will be initially tested under an “intent-to-treat” model in which data are analyzed according to treatment assignment at randomization. We will then conduct “completer analyses” on those subjects completing at least two-thirds of intervention sessions. Intervention impact will be evaluated by comparing primary study outcomes between intervention and control groups across the three time points: baseline, 6 months and 12 months post-intervention. Initially, summary scores of outcomes will be stratified by participation in the intervention versus control groups and by time point, and will be compared using chi-square tests (binary or categorical outcomes), t-tests and analysis of variance (continuous outcomes). Generalized linear mixed effects models (GLMM) accounting for within-group correlation across longitudinal data will then be applied. Outcomes will be modeled as a function of group assignment and time since intervention. The association between outcomes and time will be explored visually using scatterplots and lowess smoothers. Restricted cubic splines will be added to reflect deviations from linearity.

If siblings enter the study, they will be randomized to the same intervention group to limit contamination. In this case, analyses will be adjusted to take into account intra-sibling correlation as well. Additionally, sensitivity analyses will be conducted including and excluding siblings to examine the impact on study results. Missing data will be handled as follows: 1) identify erroneous data, 2) document reason(s) for missing and erroneous data to inform model development; 3) compare demographic characteristics of those who dropped out and those who did not to compare if data is missing at random; and 3) conduct sensitivity analysis to compare inferences that are based on different plausible reasons for missingness. Navajo and Hopkins investigators will collaborate with community partners in data interpretation to assure accuracy, cultural acceptance and relevance.

## Discussion

This study protocol presents one of the first RCTs of a program designed by and for a Native community to promote protective factors associated with delayed substance use and sexual initiation among young girls.

This study has many strengths. First, as previously noted, AB was designed in consultation with Navajo cultural specialists and was shown to be culturally appropriate and acceptable in the pilot trial (Chambers 2021). Public health interventions with roots deriving from cultural teachings have brought inclusion and participant connection with the lesson content, thus solidifying the importance of integrating culture as the foundation for prevention programs specific to AI communities. Further, the program is designed specifically for female adolescents and draws on the traditional matrilineal society of the Navajo. Across ethnicities, female adolescents have distinct patterns and processes for underage substance use and sexual risk-taking that are different from male adolescents [59, 60]. Some of these differences include females being more likely than males to use substances due to low self-esteem [61, 62], and to be offered substances in private settings by female relatives [63, 64]. Also, lack of family support is a stronger risk factor for substance use and sexual initiation among Native female adolecsents than male adolescents [62]. Thus, a gender specific approach is a strength of this program.

Second, there is substantial evidence that parental involvement in teen pregnancy and substance use prevention programs is advantageous [65–67]. However, researchers have cited the lack of parental involvement in sexual health promotion programming as a gap in efforts to prevent teen pregnancy. They conclude that by providing the parent and adolescent the same health promotion and sexual risk prevention information, the parent is informed of what the adolescent is learning and can reinforce these teachings at home [68]. Given this, inclusion of a caregiver in this program alongside the adolescent is a major strength and may enhance and help to sustain positive program impact long after the completion of the program. Next, this study will assess the impact of the program on individual-level factors of both the caregiver and adolescent. This will not only allow for the understanding of program impacts on both caregiver and child, but will also allow the study team to better understand, 1) how adolescent and caregiver outcomes and risk/protective factors are associated with each other, and 2) how changes in adolescent and/or caregiver outcomes mediate change in outcomes in the other member of the dyad.

Third, the study builds on more recent research highlighting the importance of primary prevention programs delivered during early adolescence, when adolescents are less likely to have initiated risk-taking behaviors such as substance use and sex [68, 69]. In addition, the AB program is designed to simultaneously impact co-occurring risk behaviors that impact Native adolescents and communities at disproportionate rates. If AB is efficacious at reducing both sets of risk behaviors, it will fill a gap in the literature calling for evidence based interventions (EBIs) that work across domains and are not specific to one risk behavior (e.g. risks for substance use or early sex). Programs such as AB may be particularly beneficial for Native and other under-resourced populations for whom few comprehensive EBIs exist.

This study also has limitations. First, all outcomes are measured via self-report and/or via interview. While self-report is the most widely used methodology to assess behavior change, it is not without biases. There are also potential biases in interview administration, such as… [70]. However, steps have been taken to reduce potential biases. The IEs are highly trained in evaluation administration and human subjects research and will not deliver any part of either AB program or condition non-monetary gifts, limiting potential evaluator bias. Other methods to reduce bias include: 1) all IEs will be Native women from the local community, 2) the same IE will conduct the baseline and all follow-up assessments for each dyad, 3) all interviews will be conducted in person (vs. some in person and some via telephone/video call), and 4) the most sensitive questions focused on actual behavior and behavioral intention are conducted via self-report (not interview).

Another limitation is that the follow-up time period is only 1 year. Since adolescents are 10–14 years of age when enrolling in this program, we do not expect a large proportion of adolescents to initiate substances or have sex by the 12 month follow-up timepoint. Thus, we do not expect to have adequate power to assess impact of AB on actual behaviors. However, we will have power to assess the impact of AB on behavioral intention. Studies indicate there is a strong, positive association between intention to abstain from sex and delayed sexual initiation [71]. Further, we will assess key protective factors that are associated with delayed sexual initiation and substance use among Native and non-Native adolescents [19, 23, 71].

Third, there is potential for some contamination between control and intervention groups, as this study will be conducted in a small communities where many families know each other. To limit contamination, we will individually randomize dyads and assign siblings to the same randomization group.

To our knowledge, this is the first RCT of a culturally grounded primary prevention program for teen pregnancy and substance use among female Native adolescents and their female caregivers. If AB proves efficacious, there will be a program for Native families that promotes protective factors associated with delayed substance use and sexual risk taking. Additionally, we may deepen our understanding of the relationship between adolescent and caregiver individual and family level factors, how each of these contribute to risk taking behaviors among Native adolescents, and how shifts in adolescent individual level factors influence caregiver outcomes and vice versa.

## Data Availability

Not Applicable. Only Study Co-Investigators have access to the final trial dataset. Trial results will be provided to community advisory boards and the Navajo Nation IRB through a report and presentation. The data set is owned by the Navajo Nation and will not be publicly available.

## Abbreviations

AI: American Indian
AB: Asdzáán Be’eena’(Female Pathways)
NNHRRB: Navajo Nation Human Subjects Research Review Board
Center: Center for American Indian Heallth
Native: Native American
RCT: Randomized Control Trial
FHCs: Family health coaches
IE: Independent evaluator
CAB: Community Advisory Board
GLMM: Generalized linear mixed effects models
EBIs: Evidence-based interventions
